# Repurposing a SARS-CoV-2 surveillance program for infectious respiratory diseases in a university setting

**DOI:** 10.3389/fpubh.2023.1168551

**Published:** 2023-09-01

**Authors:** Kylie L. King, Rachel Ham, Austin Smothers, Isaac Lee, Tyler Bowie, Erika Teetsel, Congyue Peng, Delphine Dean

**Affiliations:** ^1^Center for Innovative Medical Devices and Sensors (REDDI Lab), Clemson University, Clemson, SC, United States; ^2^Department of Bioengineering, Clemson University, Clemson, SC, United States

**Keywords:** SARS-CoV-2, influenza, flu, virus, surveillance testing, saliva

## Abstract

Standard multiplex RT-qPCR diagnostic tests use nasopharyngeal swabs to simultaneously detect a variety of infections, but commercially available kits can be expensive and have limited throughput. Previously, we clinically validated a saliva-based RT-qPCR diagnostic test for SARS-CoV-2 to provide low-cost testing with high throughput and low turnaround time on a university campus. Here, we developed a respiratory diagnostic panel to detect SARS-CoV-2, influenza A and B within a single saliva sample. When compared to clinical results, our assay demonstrated 93.5% accuracy for influenza A samples (43/46 concordant results) with no effect on SARS-CoV-2 accuracy or limit of detection. In addition, our assay can detect simulated coinfections at varying virus concentrations generated from synthetic RNA controls. We also confirmed the stability of influenza A in saliva at room temperature for up to 5 days. The cost of the assay is lower than standard nasopharyngeal swab respiratory panel tests as saliva collection does not require specialized swabs or trained clinical personnel. By repurposing the lab infrastructure developed for the COVID-19 pandemic, our multiplex assay can be used to provide expanded access to respiratory disease diagnostics, especially for community, school, or university testing applications where saliva testing was effectively utilized during the COVID-19 pandemic.

## 1. Introduction

Prior to the COVID-19 pandemic, influenza-like respiratory illnesses caused 935,000 infections and 179,000 hospitalizations in the United States annually (1). Although disease severity and mortality risk are greater in older adults (>65 years) (1), these respiratory illnesses have substantial morbidity in university students and were identified as the third leading cause of academic impediments at U.S. universities as recently as Fall 2019 (2–4). Additionally, influenza-like illnesses pose greater infection risk to students than university faculty or staff because of increased close interactions (5, 6). Transmission from young and healthy students to older faculty members also increases both adverse health outcomes and disease burden on the community (7, 8).

The SARS-CoV-2 pandemic substantially altered the prevalence of respiratory illnesses in all populations (9), best emphasized in the marked decrease in seasonal flu activity during the 2020–21 season (10). However, flu activity resurged during the 2021–22 season (11–13), and several U.S. universities experienced influenza A outbreaks in Fall 2021 (14, 15). Masking and social distancing measures implemented during the pandemic highly contributed to lowered flu levels during the pandemic; both pre- and post-pandemic studies show substantial effects of mask-wearing on influenza infections (16, 17). Additionally, coinfections of influenza A and SARS-CoV-2 during the pandemic resulted in increased disease severity and mortality in hospitalized patients (18, 19), though the prevalence of influenza and SARS-CoV-2 coinfections remains unclear, particularly in non-hospitalized populations. It is likely that the use of respiratory panels will increase due to the COVID-19 pandemic, and early diagnosis of both influenza and SARS-CoV-2 is critical to improve clinical outcomes with current anti-viral drug treatments. Thus, several prior research studies have recommended that multiplex panels be made advisable for patients with influenza-like symptoms (20–22). A more convenient and cost-effective test for simultaneous detection of both SARS-CoV-2 and influenza would greatly increase diagnostic and treatment options.

Standard RT-qPCR diagnostic tests that distinguish between SARS-CoV-2, influenza A, and influenza B utilize nasopharyngeal (NP) swabs (23). Commercially available kits such as the Cepheid Xpert^®^ Xpress SARS-CoV-2/Flu/RSV and the Roche cobas^®^ SARS-CoV-2 & influenza A/B only process NP swabs, cost upwards of $60 per sample, and have limited throughput (24, 25). These tests also require trained technicians and specialized equipment that is not commonly available in university or community settings. Saliva-based diagnostics are a promising alternative, allowing self-collection of samples and proving a more cost-effective option for large-scale screening programs (26, 27). Influenza viruses can be differentially detected in saliva samples via RT-qPCR (28–30) with similar sensitivity and specificity (31, 32), and a saliva-based diagnostic test for simultaneous detection of SARS-CoV-2, influenza A, influenza B, and respiratory syncytial virus (RSV) has been validated with simulated samples (33). However, to our knowledge, non-commercial assays have not been validated with patient samples. At-home saliva tests have previously been utilized in community screening programs for influenza outbreak prevention (34) and many U.S. universities implemented saliva-based population screening programs in response to the SARS-CoV-2 pandemic (35–39). Saliva pooling strategies have also provided low-cost, high throughput testing in university populations (39), though these methods are limited by lower sensitivity, particularly in areas with low viral prevalence (40, 41). Many large-scale labs established during the pandemic were dedicated exclusively to SARS-CoV-2 testing and are now under-utilized as community-wide testing is no longer in high demand (42). Though some of these centers are closing, existing equipment and personnel can be repurposed for other common respiratory illnesses to provide support for existing healthcare centers during surges, especially in rural communities and those lacking large-scale diagnostic labs.

We previously validated and implemented TigerSaliva, a cost-effective saliva-based diagnostic workflow to detect SARS-CoV-2 with capacity to process up to 10,000 samples per day (27, 43). We have expanded this diagnostic test to simultaneously detect influenza A and B alongside SARS-CoV-2 within a single saliva sample. Additionally, this multiplex assay can accurately detect coinfections across a range of simulated viral concentrations. Our work highlights the necessity for comprehensive diagnostic panels to maximize convenience and cost-effectiveness for surveillance testing.

## 2. Materials and methods

These studies involving human participants were reviewed and approved by the Clemson University Institutional Review Board (approval numbers: IRB2021-0703 and Pro00100731) (Supplementary Data Sheet 1). All patients/participants provided their written informed consent to participate in this study.

### 2.1. Respiratory virus assay conditions

Our assay contains primer and probe sets (Integrated DNA Technologies, Coralville, IA, United States) targeting the SARS-CoV-2 nucleocapsid (N) gene, influenza A (H1N1) matrix gene, influenza B non-structural protein gene (44), and internal control Hs_RPp30 gene. We have previously validated the TigerSaliva assay with the N gene and Hs_RPp30 gene (43). Each of the four probes was modified with a unique fluorophore to allow for quadruplex analysis (Table 1). Primer and probe concentrations were optimized using a matrix ranging from 50 to 750 nM to determine the maximum fluorescent range with a standard template concentration. Our assay was performed with Luna Universal One-Step RT-qPCR Kit (New England Biolabs, Ipswich MA, United States) using 4 μL of template with a final reaction volume of 20 μL. Thermocycling conditions are listed in Supplementary Data Sheet 2.

**Table 1:** Assay components.

Component	Sequence (5–3)	Fluor	Final Concentration
2019-nCoV- N1-For	GACCCCAAAATCAGCGAAAT	–	500 nM
2019-nCoV-N1-Rev	TCTGGTTACTGCCAGTTGAATCTG	–	500 nM
2019-nCoV-N1 Probe	/5FAM/ACCCCGCAT/ZEN/TACGTTTGGTGGACC/3IABkFQ	FAM	125 nM
Hs-RPP30-For	AGATTTGGACCTGCGAGCG	–	500 nM
Hs-RPP30-Rev	GAGCGGCTGTCTCCACAAGT	–	500 nM
Hs-RPP30 Probe	/5Cy5/TTCTGACCT/ZEN/GAAGGCTCTGCGCG/3IABkFQ	Cy5	125 nM
Inf A1 For	CAAGACCAATCYTGTCACCTCTGAC	–	500 nM
Inf A1 Rev	GCATTYTGGACAAAVCGTCTACG	–	500 nM
Inf A1 Probe	/5TEX615/TGCAGTCCTCGCTCACTGGGCACG/3IAbRQSp	TEXAS RED	500 nM
Inf B For	TCCTCAAYTCACTCTTCGAGCG	–	725 nM
Inf B Rev	CGGTGCTCTTGACCAAATTGG	–	725 nM
Inf B Probe	/5HEX/CCAATTCGA/ZEN/GCAGCTGAAACTGCG/3IABkFQ	HEX	500 nM

Primer and probe sequences and fluorophores are listed along 104 with the final concentration per reaction.

### 2.2. Limit of detection analysis

We used synthetic RNA (Twist Bioscience, San Francisco, CA, United States) SARS-CoV-2 control 2 (GenBank ID: MN908947.3), influenza H1N1 A/California/07/2009 (assembled genome), and influenza B/Lee/1940 (assembled genome) to determine the assay limits of detection (LoD) for each virus. We performed a 10-fold dilution series ranging from 1 ⨯ 10^6^ to 1 ⨯ 10^0^ copies/μL (cpu) in triplicate. Standard curves were generated from each RNA control and were used to find correlation coefficients and determine primer efficiencies (Supplementary Data Sheet 3).

### 2.3. Simulated coinfection evaluation

We performed pairwise combinations of influenza A, influenza B, and SARS-CoV-2 synthetic RNA (Twist Bioscience) to simulate coinfections. All mixtures had a final concentration of 10,000 cpu and each reaction was performed in triplicate with the standard assay conditions.

### 2.4. Sample collection and processing

Saliva samples (*n* = 71) were collected from patients at the university health center and SARS-CoV-2 surveillance test site during April–May 2022. Due to the logistics of testing at our university, we could not collect samples from the same location for validation of both viruses. For influenza validation, 50 saliva samples were collected from patients who were undergoing testing with either the Cepheid Xpert^®^ Xpress SARS-CoV-2/Flu/RSV test or BD Veritor Influenza A/B test at the university health center. 21 saliva samples were collected from patients tested with the TigerSaliva assay (43) (Clemson IRB2021-0703) for SARS-CoV-2 validation. Influenza B was not circulating in our community at the time of collection, so its clinical validity could not be determined. Saliva samples were stored at 4°C prior to processing.

Saliva samples were incubated with 10 μL of DNase I (Zymo Research, Irvine, CA, United States) to decrease total DNA content in the sample. RNA was manually extracted from saliva samples using the MagMAX™ Viral/Pathogen Nucleic Acid Isolation Kit (Thermo Fisher Scientific, Waltham, MA, United States) with a starting volume of 140 μL per manufacturer’s protocol. Samples were treated with 50 μg Proteinase K to decrease viscosity. Eluted RNA was quantified with a UV–Vis spectrophotometer (NanoDrop^™^, Thermo Fisher) and stored at −80°C.

### 2.5. Clinical sample evaluation

We performed the assay on extracted RNA from patient saliva samples (*n* = 71) matched with clinical results. Clinical samples were tested using a single-blind method to prevent investigator bias. Two samples were excluded from analysis due to poor amplification. We calculated the accuracy, positive percent agreement (PPA) and negative percent agreement (NPA) of our assay as described in similar studies (45, 46). 95% confidence intervals were calculated using either an exact binomial distribution or the normal approximation to the binomial distribution. A complete list of samples and relevant testing information is included in Supplementary Data Sheet 4.

### 2.6. Influenza A stability

We determined the stability of influenza A in saliva samples to mimic conditions for sample transportation. Patient saliva samples (*n* = 3) were stored at room temperature (~22°C) for 5 days and sample stability was evaluated at 1, 3, and 5 day time points. Samples were tested in triplicate using the multiplex assay described above and Ct values were compared to “fresh” (day 0).

## 3. Results

### 3.1. Analytical sensitivity and efficiencies

We evaluated the sensitivity of our multiplex RT-qPCR assay via 10-fold serial dilutions of synthetic RNA for SARS-CoV-2, influenza A, and influenza B, ranging from 4 ⨯ 10^6^ to 4 ⨯ 10^0^ cpu (Table 2). The LoD for SARS-CoV-2 was 4 gene copies/reaction, which is similar to the original TigerSaliva assay (43). The LoD for both influenza A and B was 40 gene copies/reaction, and the associated Ct values were comparable to previous validation of the primer sets (44). Primer efficiencies were calculated with the following equation: E = −1 + 10^(−1/slope)^. Efficiencies for SARS-CoV-2, influenza A, and influenza B were 98.02, 80.45, and 90.65%, respectively. *R*^2^ values for all primer sets were ≥ 0.9854.

**Table 2:** Performance of multiplex RT-qPCR assay in saliva.

Gene copies/reaction	Mean Ct values ± SD		
	SARS-CoV-2	Influenza A	Influenza B
4 × 10^6^	12.77 ± 0.34	14.02 ± 0.10	14.00 ± 0.17
4 × 10^5^	16.24 ± 0.12	17.43 ± 0.05	17.45 ± 0.07
4 × 10^4^	19.25 ± 0.11	20.81 ± 0.11	20.55 ± 0.10
4 × 10^3^	22.37 ± 0.15	24.49 ± 0.02	23.88 ± 0.16
4 × 10^2^	25.49 ± 0.41	27.94 ± 0.18	27.57 ± 0.26
4 × 10^1^	29.94 ± 0.52	**34.28 ± 1.98**	**32.24 ± 1.64**
4 × 10^0^	**34.04 ± 1.56**	39.92	nd
E	98.02%	80.45%	90.65%
*R*^2^	0.9942	0.9854	0.9949

Limits of detection for each 159 virus were evaluated using synthetic RNA and are defined as the lowest Ct value present in all 160 three replicates. Limits of detection are shown in bold. nd, not detected.

### 3.2. Clinical performance in saliva

We compared assay results from saliva samples with clinical results from Cepheid Xpert^®^ Xpress SARS-CoV-2/Flu/RSV kit or BD Veritor Influenza A/B clinical kit to determine accuracy, PPA, and NPA for the influenza A component of the assay (Table 3). Saliva samples were considered valid with duplicate amplification of the internal control gene Hs_RPp30 (Cy5 Ct < 33). Samples with duplicate amplification of matrix gene (Texas Red Ct < 30) were considered positive for influenza A (47). Samples with at least one replicate Ct value for the matrix gene above 30 were considered inconclusive and were excluded from this comparison (*n* = 4). The total accuracy of the assay for influenza A was 93.5%, PPA was 100%, and NPA was 89.7% (Supplementary Data Sheet 4).

**Table 3:** Influenza A clinical performance in saliva.

		Influenza A clinical result		Assay clinical analysis		
		Positive	Negative	% Accuracy	% PPA	% NPA
Influenza A assay result	Positive	17	0	93.5 [92.4, 94.5]	100.0 [86.8, 100]	89.7 [89.4, 89.9]
	Negative	3	26			

95% confidence interval is represented in brackets.

PPA, positive percent agreement; NPA, negative percent agreement.

In addition, we compared assay results from SARS-CoV-2 positive samples (*n* = 21) that were previously tested using the TigerSaliva assay (26, 43). Two samples were excluded due to insufficient amplification of Hs_RPp30. 18 of the 19 samples were positive for SARS-CoV-2 on this multiplex assay, yielding 94.7% accuracy for detection of SARS-CoV-2. As identified through the SARS-CoV-2 sequencing program at Clemson, the variants circulating on the university campus at the time were all (100%) Omicron variants with the predominant subvariant being BA.2 (50.6%) and associated sub-variants (All BA.2 and BA.2 sub-variants: 91.7%).

### 3.3. Simulated coinfection evaluation

**Figure 1:**
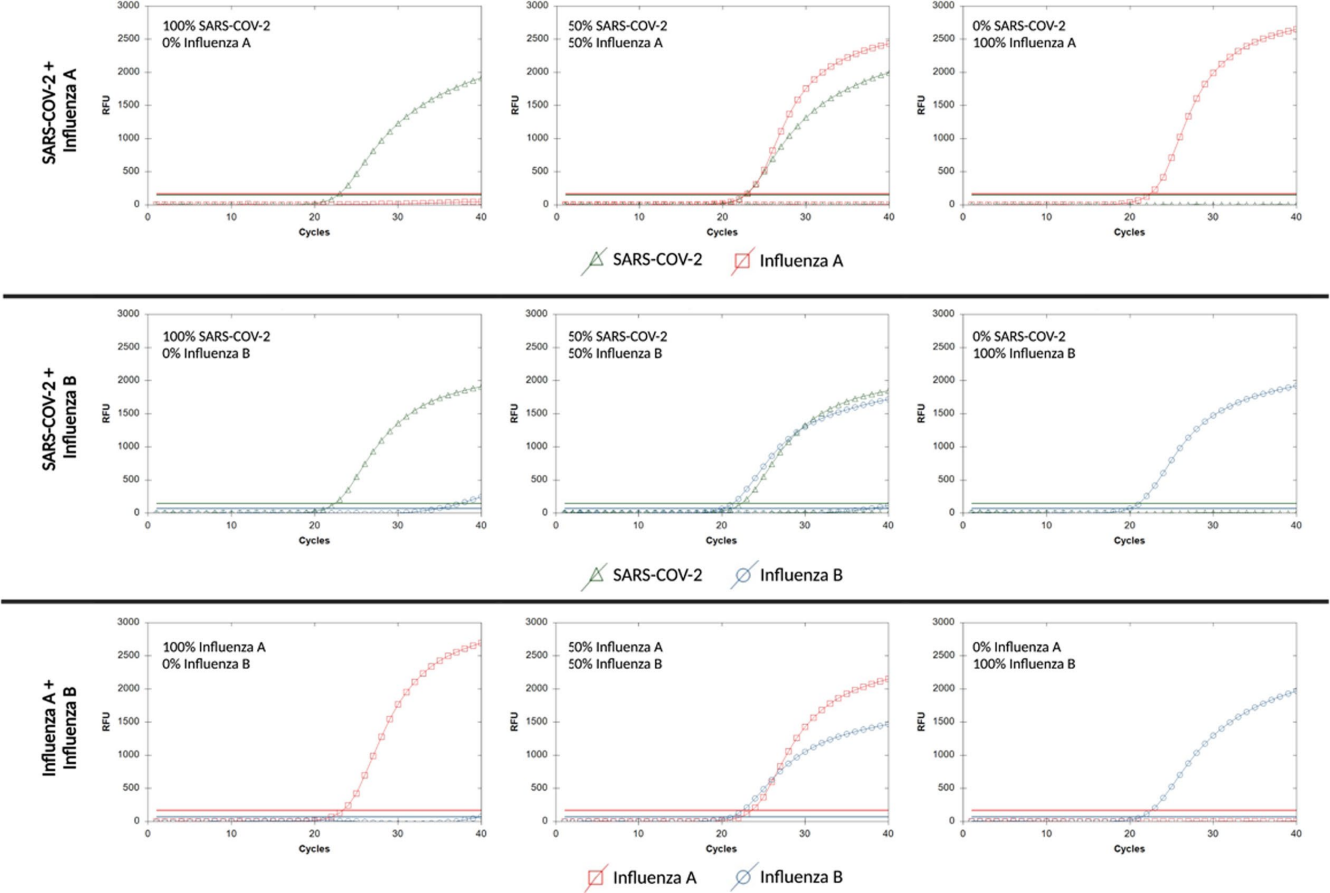
Representative curves of synthetic viral RNA mixtures. Synthetic positive controls 192 for each virus were mixed to evaluate assay performance in the presence of two viruses. 193 representative curves are shown for each combination.

We mixed synthetic controls for SARS-CoV-2, influenza A, and influenza B to simulate coinfections (Figure 1). Synthetic controls were run individually at a concentration of 10,000 cpu and in pairwise combinations with final concentrations of 5,000 cpu for each virus. The assay accurately detected both viruses in each mixture within the same reaction. Small amounts of non-specific amplification from the FAM probe were observed in the 100% SARS-CoV-2 reaction (Ct > 35, RFU < 600) (Figure 1).

SARS-CoV-2 is known to be stable in saliva samples at room temperature (26), but the stability of influenza is yet to be investigated. To evaluate influenza A stability in saliva, we stored samples (*n* = 3) at room temperature and tested using the assay on days 0, 1, 3, and 5. We found that influenza A is stable in saliva for up to 5 days with only minimal degradation (average increase of 3.4 cycles). We were not able to obtain influenza B samples, so its stability in saliva could not be determined.

## 4. Discussion

We expanded upon the pre-existing SARS-CoV-2 surveillance protocol at our university to simultaneously detect influenza A and B in saliva samples. Our multiplex assay shows that influenza A and B synthetic RNA can be detected without affecting SARS-CoV-2 detection. Influenza A virus can also be detected in patient saliva samples with 93.5% accuracy using our assay (Table 3). To our knowledge, we are the first group to validate a non-commercial multiplex RT-qPCR assay with influenza A patient samples. We determined that a Ct cutoff of 30 for all targets maximized assay precision while reducing likelihood of false positive results based on experimental analytical sensitivity (47). Additionally, there was no decrease in primer efficiency for SARS-CoV-2 detection in the multiplex assay as compared to the original TigerSaliva assay (48). We observed that influenza A primer efficiency in saliva was lower than that of SARS-CoV-2 and influenza B (Table 3), though no efficiencies were calculated by other groups utilizing the same primer set for traditional swab samples (44).

We determined that our assay can also detect coinfections of SARS-CoV-2, influenza A, or influenza B at varying ratios of synthetic viral concentration (Figure 1). Reports of coinfections in a clinical setting are scarce and little is known about the relative quantities of pathogens within coinfections (49–52). Thus, one benefit of our assay is that multiple infections can be detected independently of individual virus concentrations. We observed a small amount of signal bleed over in the FAM channel for influenza B (Figure 1), which may be caused by the overlap of absorption spectra for both FAM and HEX fluorophores. We attempted to compensate for this by adjusting primer and probe ratios, but this did not decrease the level of background signal.

One limitation of this study was the lack of clinical influenza B samples. Influenza B was not circulating in our community during the study, and we were unable to obtain patient samples. In addition, influenza B levels in the U.S. were considerably lower than influenza A for the 2022–2023 flu season, accounting for less than 2% of all flu cases reported (13). One strain of influenza B is believed to have gone extinct during the COVID-19 pandemic (53). However, it should be noted that cultured influenza B virus has previously been measured in patient saliva with high clinical sensitivity and selectivity (90–100%) (31, 54).

**Figure 2:**
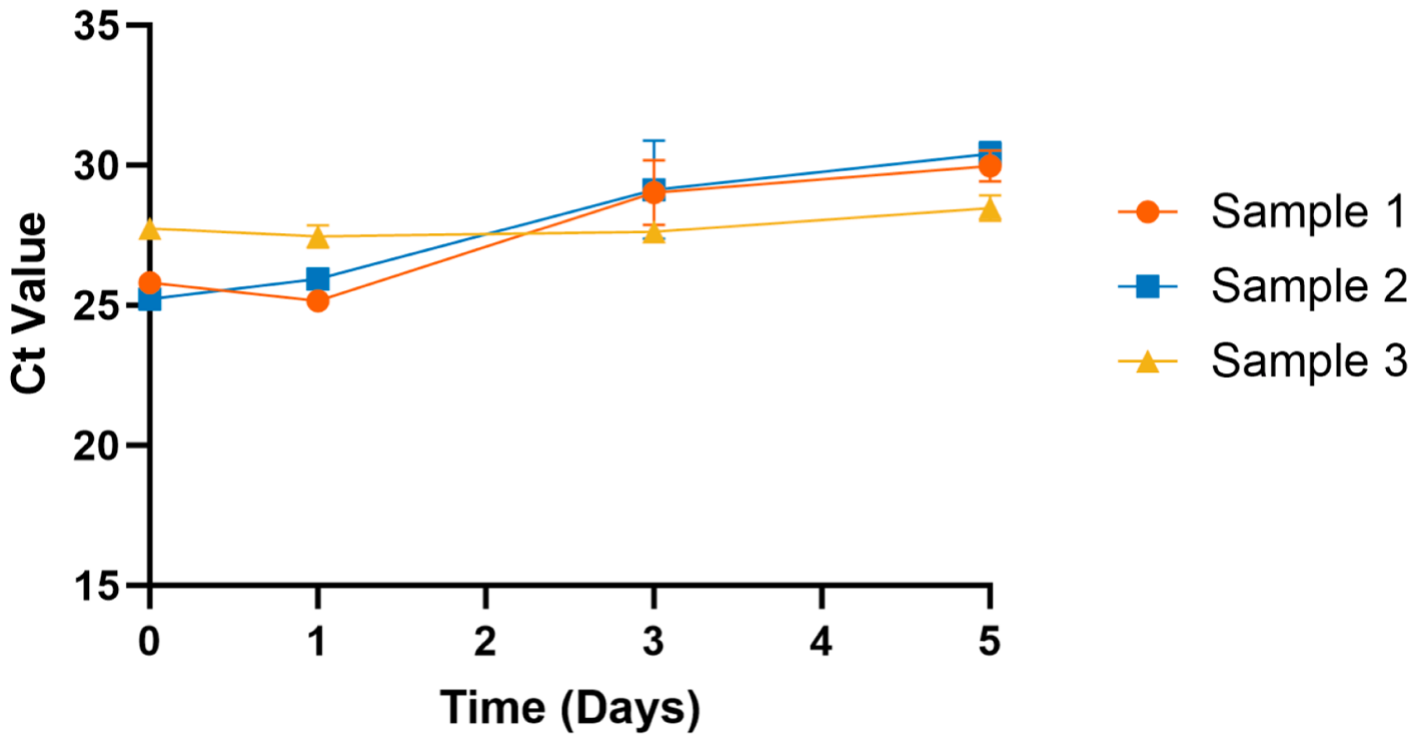
Stability of influenza A in saliva at room temperature. Influenza A is stable in saliva samples at room temperature (~20°C) for 5 days without addition of any stabilization buffers. Ct values increased slightly at days 3 and 5 but remained within detectable limits (Ct < 30).

Prior studies have shown that additives or stabilization buffers are not required to prevent SARS-CoV-2 viral degradation in saliva (26, 55). Here, we have confirmed that influenza A can be reliably detected in saliva samples for 5 days at room temperature without the addition of stabilizing buffers (Figure 2). This allows for greater flexibility in sample transportation to the laboratory and can expand access to testing in rural areas. The main advantage of our assay is multiple diagnostic targets within the same high-throughput, low-cost test. We estimate the cost of this assay to be similar to low-cost COVID-19 saliva tests (<$10/test) used during the pandemic, such as our own TigerSaliva assay (43) or the SalivaDirect assay (26). Other cost-lowering measures include sample pooling, which greatly reduces reagents needed, but can increase the chances of false negatives due to the lack of internal control for each sample. Sensitivity of sample pooling decreases further when the community prevalence of the viral target is low, as it was for influenza during the SARS-CoV-2 pandemic (40, 41).

Limitations to this study included a small sample size and samples collected from two different locations, due to constraints around sample collection, which may weaken sensitivity and specificity calculations (56). Future studies should focus on collecting a higher number of influenza A and B samples for further clinical validation. Additionally, pre- and post-analytic factors should be considered, particularly for scale up and large-scale community testing. Self-collection of samples inherently introduces more error, though steps were taken to provide thorough instructions to all participants. In our prior experience of large scale COVID-19 saliva surveillance (26, 43, 48) with over 1.1 million saliva tests collected, less than 0.15% of samples were deemed invalid due to broken collection tubes, visible food particles, or other issues which prevented the samples from being analyzed. In addition, less than 0.3% of samples returned inconclusive results due to low-quality amplification of the Hs_RPp30 control. Altogether, saliva is a relatively robust sample type and can be reliably self-collected for large community testing applications. Expanding this saliva testing for other respiratory diseases could facilitate community testing programs, especially in places that do not have ready access to health care professionals to collect the standard swab assays, such as schools, universities, or community outreach programs.

## 5. Conclusion

SARS-CoV-2 saliva-based testing can be multiplexed to screen for influenza, which may increase voluntary testing and reduce the burden on community health services and external healthcare providers. In addition, influenza A and SARS-CoV-2 are stable in saliva over several days in ambient conditions without the need for specialized buffers or stabilizers, which facilitates easy transport to laboratories for testing. Accessible testing programs have the potential to decrease the spread of disease in settings where standard clinical collection of samples is not feasible. Thus, this multiplex assay can expand the capabilities of the community and university testing labs that were established during the pandemic and allow broader surveillance of diseases beyond COVID-19.

## Data availability statement

The original contributions presented in the study are included in the article/Supplementary material, further inquiries can be directed to the corresponding authors.

## Ethics statement

The studies involving humans were approved by Clemson University, Prisma Health. The studies were conducted in accordance with the local legislation and institutional requirements. The participants provided their written informed consent to participate in this study.

## Author contributions

KK, RH, and DD conceptualized the manuscript. RH, KK, and CP designed project methodology. RH, KK, IL, TB, and ET performed the experiments. KK and RH mentored and instructed Creative Inquiry undergraduate students. KK, AS, and RH curated, analyzed, presented data, and drafted the manuscript. KK, RH, AS, CP, and DD contributed to close analysis and editing of the manuscript. All authors contributed to read and approved the submitted manuscript.

## Funding

This project was funded by SC Governor and Joint Bond Review Committee, NIH NIGMS 3P20GM121342-03S1, NIH U01AA029328, NIH P20GM139769, and Clemson University Office of Creative Inquiry and Undergraduate Research.

## Conflict of interest

The authors declare that the research was conducted in the absence of any commercial or financial relationships that could be construed as a potential conflict of interest.

## Publisher’s note

All claims expressed in this article are solely those of the authors and do not necessarily represent those of their affiliated organizations, or those of the publisher, the editors and the reviewers. Any product that may be evaluated in this article, or claim that may be made by its manufacturer, is not guaranteed or endorsed by the publisher.

## Supplementary material

SUPPLEMENTARY DATA SHEET 4 Sample Identification Sheet.

## References

[1] Troeger CE Blacker BF Khalil IA Zimsen SRM Albertson SB Abate D. Mortality, morbidity, and hospitalisations due to influenza lower respiratory tract infections, 2017: an analysis for the Global Burden of Disease Study 2017. Lancet Respir Med. (2019) 7:69–89. doi: 10.1016/S2213-2600(18)30496-X, PMID: 30553848 PMC6302221

[2] American College Health Association. Undergraduate student reference group data report fall 2019. Silver Spring, MD: American College Health Association (2020).

[3] Nichol KL D’Heilly S Ehlinger E. Colds and influenza-like illnesses in university students: impact on health, academic and work performance, and health care use. Clin Infect Dis. (2005) 40:1263–70. doi: 10.1086/429237/2/, PMID: 15825028

[4] Mullins J Cook R Rinaldo C Yablonsky E Hess R Piazza P. Influenza-like illness among university students: symptom severity and duration due to influenza virus infection compared to other etiologies. J Am Coll Heal. (2011) 59:246–51. doi: 10.1080/07448481.2010.502197, PMID: 21308583 PMC4944816

[5] Iuliano AD Reed C Gun A Desai M Dee DL Kutty P. Notes from the field: outbreak of 2009 pandemic influenza A (H1N1) virus at a large public University in Delaware, April-May 2009. Clin Infect Dis. (2009) 49:1811–20. doi: 10.1086/649555/2/, PMID: 19911964

[6] Davis BM Foxman B Monto AS Baric RS Martin ET Uzicanin A. Human coronaviruses and other respiratory infections in young adults on a university campus: prevalence, symptoms, and shedding. Influenza Other Respir Viruses. (2018) 12:582–90. doi: 10.1111/irv.12563, PMID: 29660826 PMC6086849

[7] Wu Z Harrich D Li Z Hu D Li D. The unique features of SARS-CoV-2 transmission: comparison with SARS-CoV, MERS-CoV and 2009 H1N1 pandemic influenza virus. Rev Med Virol. (2021) 31:e2171. doi: 10.1002/rmv.2171, PMID: 33350025 PMC7537046

[8] Hsieh YH. Age groups and spread of influenza: implications for vaccination strategy. BMC Infect Dis. (2010) 10:1–12. doi: 10.1186/1471-2334-10-106, PMID: 20429954 PMC2876165

[9] Uhteg K Amadi A Forman M Mostafa HH. Circulation of non-SARS-CoV-2 respiratory pathogens and coinfection with SARS-CoV-2 amid the COVID-19 pandemic. Infect Dis. (2022) 9:ofab618. doi: 10.1093/ofid/ofaB618 PMC8863080 35211632

[10] Olsen SJ Winn AK Budd AP Prill MM Steel J Midgley CM. Changes in influenza and other respiratory virus activity during the COVID-19 pandemic — United States, 2020–2021. MMWR Morb Mortal Wkly Rep. (2021) 70:1013–9. doi: 10.15585/mmwr.mm7029A1, PMID: 34292924 PMC8297694

[11] Bancej C Rahal A Lee L Buckrell S Schmidt K Bastien N. National FluWatch mid-season report, 2021-2022: sporadic influenza activity returns. Can Commun Dis Rep. (2022) 48:39–45. doi: 10.14745/ccdr.v48i01a06, PMID: 35273468 PMC8856831

[12] Chung JR Kim SS Kondor RJ Smith C Budd AP Tartof SY. Interim estimates of 2021–22 seasonal influenza vaccine effectiveness — United States, February 2022. MMWR Morb Mortal Wkly Rep. (2022) 71:365–70. doi: 10.15585/mmwr.mm7110A1, PMID: 35271561 PMC8911998

[13] Centers for Disease Control. Weekly U.S. influenza surveillance report | CDC. CDC surveillance reports. (2023). Available at: https://www.cdc.gov/flu/weekly/index.htm (Accessed February 5, 2023)

[14] Delahoy MJ Mortenson L Bauman L Marquez J Bagdasarian N Coyle J. Influenza A(H3N2) outbreak on a university campus — Michigan, October–November 2021. MMWR Morb Mortal Wkly Rep. (2021) 70:1712–4. doi: 10.15585/mmwr.mm7049E1, PMID: 34882659 PMC8659183

[15] Whitford E. Flu virus grips college campuses Inside Higher Education (2021). Available at: https://www.insidehighered.com/news/2021/11/17/flu-virus-grips-college-campuses

[16] Brienen NCJ Timen A Wallinga J Van Steenbergen JE Teunis PFM. The effect of mask use on the spread of influenza during a pandemic. Risk Anal. (2010) 30:1210–8. doi: 10.1111/j.1539-6924.2010.01428.x, PMID: 20497389 PMC7169241

[17] Froese H A Prempeh AG. Mask use to curtail influenza in a post-COVID-19 world: modeling study. JMIRx Med. (2022) 3:e31955. doi: 10.2196/31955, PMID: 35666696 PMC9153293

[18] Alosaimi B Naeem A Hamed ME Alkadi HS Alanazi T Al Rehily SS. Influenza co-infection associated with severity and mortality in COVID-19 patients. Virol J. (2021) 18:127–9. doi: 10.1186/S12985-021-01594-0, PMID: 34127006 PMC8200793

[19] Zheng J Chen F Wu K Wang J Li F Huang S. Clinical and virological impact of single and dual infections with influenza A (H1N1) and SARS-CoV-2 in adult inpatients. PLoS Negl Trop Dis. (2021) 15:e0009997. doi: 10.1371/journal.pntd.0009997, PMID: 34843492 PMC8659415

[20] Krumbein H Kümmel LS Fragkou PC Thölken C Hünerbein BL Reiter R. Respiratory viral co-infections in patients with COVID-19 and associated outcomes: a systematic review and meta-analysis. Rev Med Virol. (2023) 33:e2365. doi: 10.1002/rmv.2365, PMID: 35686619 PMC9347814

[21] Popowitch EB Kaplan S Wu Z Tang YW Miller MB. Comparative performance of the luminex NxTAG respiratory pathogen panel, GenMark eSensor respiratory viral panel, and BioFire FilmArray respiratory panel. Microbiol Spectr. (2022) 10:e0124822. doi: 10.1128/spectrum.01248-22, PMID: 35766513 PMC9431521

[22] Petrocelli PA Cunsolo V Melito M Scuderi G Testa R Messina S. Diagnosis of respiratory syncytial virus (RSV) infection in children by respiratory panel utilized during the COVID-19 pandemic. Ann Ist Super Sanita. (2023) 59:31–6. doi: 10.4415/ANN_23_01_05, PMID: 36974702

[23] Mostafa HH Carroll KC Hicken R Berry GJ Manji R Smith E. Multicenter evaluation of the cepheid xpert xpress SARS-CoV-2/Flu/RSV test. J Clin Microbiol. (2021) 59:e02955-20. doi: 10.1128/JCM.02955-20, PMID: 33298613 PMC8106732

[24] Cepheid. Cepheid | Cepheid | Xpert^®^ Xpress SARS-CoV-2/Flu/RSV (EUA). (2023). Available at: https://www.cepheid.com/en_US/tests/Critical-Infectious-Diseases/Xpert-Xpress-SARS-CoV-2-Flu-RSV (Accessed February 5, 2023).

[25] Roche. cobas^®^ SARS-CoV-2 & influenza A/B assay. (2023). Available at: https://diagnostics.roche.com/us/en/products/params/cobas-sars-cov-2-influenza-a-b-nucleic-acid-test.html (Accessed February 5, 2023).

[26] Vogels CBF Watkins AE Harden CA Brackney DE Shafer J Wang J. SalivaDirect: a simplified and flexible platform to enhance SARS-CoV-2 testing capacity. Med. (2021) 2:263–280.e6. doi: 10.1016/j.medj.2020.12.010, PMID: 33521748 PMC7836249

[27] Plumb EV Ham RE Napolitano JM King KL Swann TJ Kalbaugh C. Implementation of a rural community diagnostic testing strategy for SARS-CoV-2 in upstate South Carolina. Front Public Health. (2022) 10:858421. doi: 10.3389/fpubh.2022.858421, PMID: 35450120 PMC9016164

[28] Van Elden LJR Nijhuis M Schipper P Schuurman R Van Loon AM. Simultaneous detection of influenza viruses A and B using real-time quantitative PCR. J Clin Microbiol. (2001) 39:196–200. doi: 10.1128/JCM.39.1.196-200.2001/ASSET/76B6DCBA-92CD-43D7-8252-8D0B01867813, PMID: 11136770 PMC87701

[29] Kim YG Yun SG Kim MY Park K Cho CH Yoon SY. Comparison between saliva and nasopharyngeal swab specimens for detection of respiratory viruses by multiplex reverse transcription-PCR. J Clin Microbiol. (2016) 55:226–33. doi: 10.1128/JCM.01704-16, PMID: 27807150 PMC5228234

[30] Zhou B Deng YM Barnes JR Sessions OM Chou TW Wilson M. Multiplex reverse transcription-PCR for simultaneous surveillance of influenza A and B viruses. J Clin Microbiol. (2017) 55:3492–501. doi: 10.1128/JCM.00957-17, PMID: 28978683 PMC5703814

[31] Galar A Catalán P Vesperinas L Miguens I Muñoz I García-Espona A. Use of saliva swab for detection of influenza virus in patients admitted to an emergency department. Microbiol Spectr. (2021) 9:e0033621. doi: 10.1128/Spectrum.00336-21, PMID: 34431684 PMC8552598

[32] Sueki A Matsuda K Yamaguchi A Uehara M Sugano M Uehara T. Evaluation of saliva as diagnostic materials for influenza virus infection by PCR-based assays. Clin Chim Acta. (2016) 453:71–4. doi: 10.1016/J.CCA.2015.12.006, PMID: 26656311

[33] Sahajpal NS Mondal AK Ananth S Njau A Jones K Ahluwalia P. Clinical validation of a multiplex PCR-based detection assay using saliva or nasopharyngeal samples for SARS-CoV-2, influenza A and B. Sci Rep. (2022) 12:3480–7. doi: 10.1038/s41598-022-07152-0, PMID: 35241679 PMC8894395

[34] Goff J Rowe A Brownstein JS Chunara R. Surveillance of acute respiratory infections using community-submitted symptoms and specimens for molecular diagnostic testing. PLoS Curr Outbreaks. (2015) 7. doi: 10.1371/currents.outbreaks.0371243baa7f3810ba1279e30b96d3b6, PMID: 26075141 PMC4455990

[35] Avendano C Lilienfeld A Rulli L Stephens M Barrios WA Sarro J. SARS-CoV-2 variant tracking and mitigation during in-person learning at a midwestern university in the 2020-2021 school year. JAMA Netw Open. (2022) 5:e2146805. doi: 10.1001/jamanetworkopen.2021.46805, PMID: 35113163 PMC8814910

[36] Rennert L Kalbaugh CA McMahan C Shi L Colenda CC. The impact of phased university reopenings on mitigating the spread of COVID-19: a modeling study. BMC Public Health. (2021) 21:1520. doi: 10.1186/S12889-021-11525-X, PMID: 34362333 PMC8343346

[37] Ehrenberg AJ Moehle EA Brook CE Cate AHD Witkowsky LB Sachdeva R. Launching a saliva-based SARS-CoV-2 surveillance testing program on a university campus. PLoS One. (2021) 16:e0251296. doi: 10.1371/journal.pone.0251296, PMID: 34038425 PMC8153421

[38] Ranoa DRE Holland RL Alnaji FG Green KJ Wang L Fredrickson RL. Mitigation of SARS-CoV-2 transmission at a large public university. Nat Commun. (2022) 13:3207. doi: 10.1038/s41467-022-30833-3 35680861 PMC9184485

[39] Vander Schaaf NA Fund AJ Munnich BV Zastrow AL Fund EE Senti TL. Routine, cost-effective SARS-CoV-2 surveillance testing using pooled saliva limits viral spread on a residential college campus. Microbiol Spectr. (2021) 9:e0108921. doi: 10.1128/Spectrum.01089-21, PMID: 34643445 PMC8515933

[40] Moreno-Contreras J Espinoza MA Sandoval-Jaime C Cantú-Cuevas MA Madrid-González DA Barón-Olivares H. Pooling saliva samples as an excellent option to increase the surveillance for SARS-CoV-2 when re-opening community settings. PLoS One. (2022) 17:e0263114. doi: 10.1371/journal.pone.0263114, PMID: 35077513 PMC8789121

[41] Pasomsub E Watcharananan SP Watthanachockchai T Rakmanee K Tassaneetrithep B Kiertiburanakul S. Saliva sample pooling for the detection of SARS-CoV-2. J Med Virol. (2021) 93:1506–11. doi: 10.1002/jmv.26460, PMID: 32841429 PMC7461487

[42] Das S Frank KM. Strategies for scaling up SARS-CoV-2 molecular testing capacity. Clin Lab Med. (2022) 42:261–82. doi: 10.1016/j.cll.2022.02.006, PMID: 35636826 PMC8901375

[43] Ham RE Smothers AR King KL Napalitano JM Swann TJ Pekarek LG. Efficient SARS-CoV-2 quantitative reverse transcriptase PCR saliva diagnostic strategy utilizing open-source pipetting robots. J Vis Exp. (2022) 180:e63395. doi: 10.3791/63395, PMID: 35225290 PMC9199378

[44] Shu B Kirby MK Davis WG Warnes C Liddell J Liu J. Multiplex real-time reverse transcription PCR for influenza A virus, influenza B virus, and severe acute respiratory syndrome coronavirus 2 - volume 27, number 7—July 2021 - emerging infectious diseases journal - CDC. Emerg Infect Dis. (2021) 27:1821–30. doi: 10.3201/EID2707.210462, PMID: 34152951 PMC8237866

[45] Ham RE Smothers AR Che R Sell KJ Peng CA Dean D. Identifying SARS-CoV-2 variants of concern through saliva-based RT-qPCR by targeting recurrent mutation sites. Microbiol Spectr. (2022) 10:e0079722. doi: 10.1128/spectrum.00797-22, PMID: 35546574 PMC9241879

[46] Parikh R Mathai A Parikh S Sekhar GC Thomas R. Understanding and using sensitivity, specificity and predictive values. Indian J Ophthalmol. (2008) 56:45–50. doi: 10.4103/0301-4738.37595, PMID: 18158403 PMC2636062

[47] Caraguel CGB Stryhn H Gagné N Dohoo IR Hammell KL. Selection of a cutoff value for real-time polymerase chain reaction results to fit a diagnostic purpose: analytical and epidemiologic approaches. J Vet Diagn. (2011) 23:2–15. doi: 10.1177/104063871102300102, PMID: 21217022

[48] King KL Wilson S Napolitano JM Sell KJ Rennert L Parkinson CL. SARS-CoV-2 variants of concern alpha and delta show increased viral load in saliva. PLoS One. (2022) 17:e0267750. doi: 10.1371/journal.pone.0267750, PMID: 35536777 PMC9089873

[49] Malekifar P Pakzad R Shahbahrami R Zandi M Jafarpour A Rezayat SA. Viral coinfection among COVID-19 patient groups: an update systematic review and meta-analysis. Biomed Res Int. (2021) 2021:1–10. doi: 10.1155/2021/5313832, PMID: 34485513 PMC8416381

[50] Fujita DM dos Santos SG Sartori GP da Silva H Nali L. COVID-19 and influenza coinfection: the rise of Ômicron and H3N2 in Brazil – 2022. Travel Med Infect Dis. (2022) 46:102262. doi: 10.1016/J.TMAID.2022.102262, PMID: 35038569 PMC8759102

[51] Ozaras R Cirpin R Duran A Duman H Arslan O Bakcan Y. Influenza and COVID-19 coinfection: report of six cases and review of the literature. J Med Virol. (2020) 92:2657–65. doi: 10.1002/JMV.26125, PMID: 32497283

[52] Yue H Zhang M Xing L Wang K Rao X Liu H. The epidemiology and clinical characteristics of co-infection of SARS-CoV-2 and influenza viruses in patients during COVID-19 outbreak. J Med Virol. (2020) 92:2870–3. doi: 10.1002/JMV.26163, PMID: 32530499 PMC7307028

[53] Vajo Z Torzsa P. Extinction of the influenza B Yamagata line during the COVID pandemic-implications for vaccine composition. Viruses. (2022) 14:1745. doi: 10.3390/v14081745, PMID: 36016367 PMC9414795

[54] Neopane P Nypaver J Shrestha R Beqaj S. Performance evaluation of TaqMan SARS-CoV-2, Flu A/B, RSV RT-PCR multiplex assay for the detection of respiratory viruses. Infect Drug Resist. (2022) 15:5411–23. doi: 10.2147/IDR.S373748, PMID: 36119638 PMC9480588

[55] Ott IM Strine MS Watkins AE Boot M Kalinich CC Harden CA. Stability of SARS-CoV-2 RNA in nonsupplemented saliva. Emerg Infect Dis. (2021) 27:1146–50. doi: 10.3201/eid2704.204199, PMID: 33754989 PMC8007305

[56] Bujang MA Adnan TH. Requirements for minimum sample size for sensitivity and specificity analysis. J Clin Diagn Res. (2016) 10:YE01–6. doi: 10.7860/JCDR/2016/18129.8744, PMID: 27891446 PMC5121784

