# Supplementary material for: Repurposing a SARS-CoV-2 surveillance program for infectious respiratory diseases in a university setting
Source: Front Public Health. 2023 Sep 1;11:1168551. doi: 10.3389/fpubh.2023.1168551 (PMC10505707; doi:10.3389/fpubh.2023.1168551)
Supplement: Supplementary file 2 [file Data_Sheet_1.pdf]

Division of
RESEARCH
Research Compliance

To: Delphine Dean
Re: Clemson IRB Number: IRB2021-0703

Review Level:	Expedited
Determination Date:	01-Dec-2021
Completion Date:	31-May-2022
Funding Sponsor	N/A
Project Title:	Saliva Samples for Clinical Validation of Multiplex Assay

---

The Clemson University Institutional Review Board (IRB) determined that the proposed activities involving human participants meet the criteria for expedited review under 45 CFR 46.111.

Informed Consent: Study personnel are required to use the approved informed consent document(s) for the study, unless a waiver of consent is granted.

Informed consent waiver(s) granted for this study: No waivers were granted.

Principal Investigator (PI) Responsibilities: The PI assumes the responsibilities for the protection of human subjects as outlined in the Principal Investigator's Responsibilities guidance.

Non-Clemson Affiliated Collaborators: This determination only covers Clemson affiliated researchers onthe study. External collaborators will have to consult with their respective institution's IRB office to determine what is required for their role on the project. An IRB Authorization Agreement is required for Clemson's IRB to be the IRB record for the study.

Progress Report: A progress report is required at least 30 days before the scheduled expiration date to extend the approval period.

Modifications: The PI is required to submit all proposed changes (i.e., increase in enrollment; modifications to research methods/instruments, recruitment procedures/documents, incentives, informed consent process/document, data, etc.) to the IRB office using the amendment request form. All changes must be reviewed and approved prior to implementation; except when an immediate change is necessary to eliminate a hazard to the participants, or to provide

participants with new information on adverse event or research results considered essential to a participant's decision whether to continue participation.

New Funding: Notify the IRB office If new funding is received for an active study. IRB review of the new award must be completed before new funds can be spent on human research activities, as the new funding source may have additional or different requirements.

Reportable Events: Notify the IRB office immediately if there are any unanticipated problems involving risk to subjects, complications, adverse events and/or any complaints from research participants that maychange the level of review from exempt to expedited or full board review.

Study Personnel Changes: An amendment request form is required for all personnel changes. CITI training documentation is required for all team members, including the PI.

CITI Training: All study personnel are required to complete the CITI human subjects training course.

Non-Clemson Affiliated Sites: A site letter is required for off-campus sites. Refer to the guidance on research site/permission letters for more information. An amendment request form is required to add additional sites to the study.

International Research: Clemson's approval is based on U.S. human subjects protections regulations and Clemson University human subjects protection policies. Researchers should become familiar with all pertinent information about local human subjects protection regulations and requirements when conducting research in countries other than the United States. We encourage you to discuss with your local contacts any possible human subjects research requirements that are specific to your research site, to comply with those requirements and to inform Clemson's IRB office of those requirements so we can better help other researchers prepare for international research in the future.

New IRB Application: A new application is required if the study remains open for more than 5 years after the initial determination.

Closure: Notify the IRB office when the study can be closed or if the PI leaves the university. Closure indicates that research activities with human subjects are no longer ongoing, have stopped and are complete. Human research activities are complete when investigators are no longer obtaining information or biospecimens about a living person through interaction or intervention with the individual, obtaining identifiable private information or identifiable biospecimens about a living person, and/or using, studying, analyzing, or generating identifiable private information or identifiable biospecimens about a living person.

Contact Information: Please contact the IRB office at IRB@clemson.edu or visit our webpage if you have questions.

Clemson University IRB is committed to facilitating ethical research and protecting the rights of human subjects. Please contact us if you have any questions and use the IRB number and title when referencing the study in future correspondence.

Institutional Review Board
Office of Research Compliance
Clemson University
https://www.clemson.edu/research/compliance/irb/

IRB Number: IRB00000481
FWA Number: FWA00004497

DATE: June 24, 2022

TO: Delphine Dean
FROM: Prisma Health Committee A

PROJECT TITLE: [1852976-2] Development of saliva based detection assays for the SARS Cov2 virus and antibodies
REFERENCE #: Pro00100731
SUBMISSION TYPE: Continuing Review/Progress Report

ACTION: APPROVED
APPROVAL DATE: June 24, 2022
EXPIRATION DATE: None - Next Report Due Date June 23, 2025
REVIEW TYPE: Expedited Review

The following items are approved in this submission:

- Consent Form - Fall2021ConsentForm.pdf (UPLOADED: 05/31/2022)
- Cover Sheet - CR Cover Sheet_ SalivaStudy06202022.docx (UPLOADED: 06/23/2022)
- Prisma Health - IRB Application - Prisma Health - IRB Application (UPLOADED: 06/20/2022)

Thank you for your submission of Continuing Review/Progress Report materials for this project. The Prisma Health Committee A has APPROVED your submission. This approval is based on an appropriate risk/benefit ratio and a project design wherein the risks have been minimized. All research must be conducted in accordance with this approved submission.

This submission has received Expedited Review based on applicable federal regulations.

Please remember that informed consent is a process beginning with a description of the project and insurance of participant understanding followed by a signed consent form. Informed consent must continue throughout the project via a dialogue between the researcher and research participant. Federal regulations require that each participant receives a copy of the consent document.

Please note that any revision to previously approved materials must be approved by this committee prior to initiation. Please use the appropriate revision forms for this procedure.

All UNANTICIPATED PROBLEMS involving risks to subjects or others (UPIRSOs) and SERIOUS and UNEXPECTED adverse events must be reported promptly to this office. Please use the appropriate reporting forms for this procedure. All FDA and sponsor reporting requirements should also be followed.

All NON-COMPLIANCE issues or COMPLAINTS regarding this project must be reported promptly to this office.

This project has been determined to be a MINIMAL RISK project. Based on the risks, this project does not require annual continuing review. The next report due date for this project will be June 23, 2025.

Generated on IRBNet

Please note that all research records must be retained for a minimum of three years after the completion of the project.

If you have any questions, please contact Prisma Health IRB at 864-455-8997 or IRB@PrismaHealth.org. Please include your project title and reference number in all correspondence with this committee.

This letter has been electronically signed in accordance with all applicable regulations, and a copy is retained within Prisma Health Committee A's records.
