# Supplementary material for: Repurposing a SARS-CoV-2 surveillance program for infectious respiratory diseases in a university setting
Source: Front Public Health. 2023 Sep 1;11:1168551. doi: 10.3389/fpubh.2023.1168551 (PMC10505707; doi:10.3389/fpubh.2023.1168551)
Supplement: Supplementary file 3 [file Data_Sheet_2.docx]

**Thermocycling Conditions.**

| **Stage** | **Temperature (°C)** | **Duration** | **Number of Cycles** |
| --- | --- | --- | --- |
| Reverse Transcription | 55 | 10 min | 1 |
| Initial Denaturation | 95 | 1 min | 1 |
| Touchdown | 95 | 10 sec | 3 |
|  | 72 | 30 sec |  |
|  | 95 | 10 sec | 3 |
|  | 69 | 30 sec |  |
|  | 95 | 30 sec | 3 |
|  | 66 | 30 sec |  |
| Main Amplification | 95 | 10 sec | 40 |
|  | 65 | 30 sec |  |
