# Supplementary material for: Repurposing a SARS-CoV-2 surveillance program for infectious respiratory diseases in a university setting
Source: Front Public Health. 2023 Sep 1;11:1168551. doi: 10.3389/fpubh.2023.1168551 (PMC10505707; doi:10.3389/fpubh.2023.1168551)
Supplement: Supplementary file 4 [file Data_Sheet_3.docx]

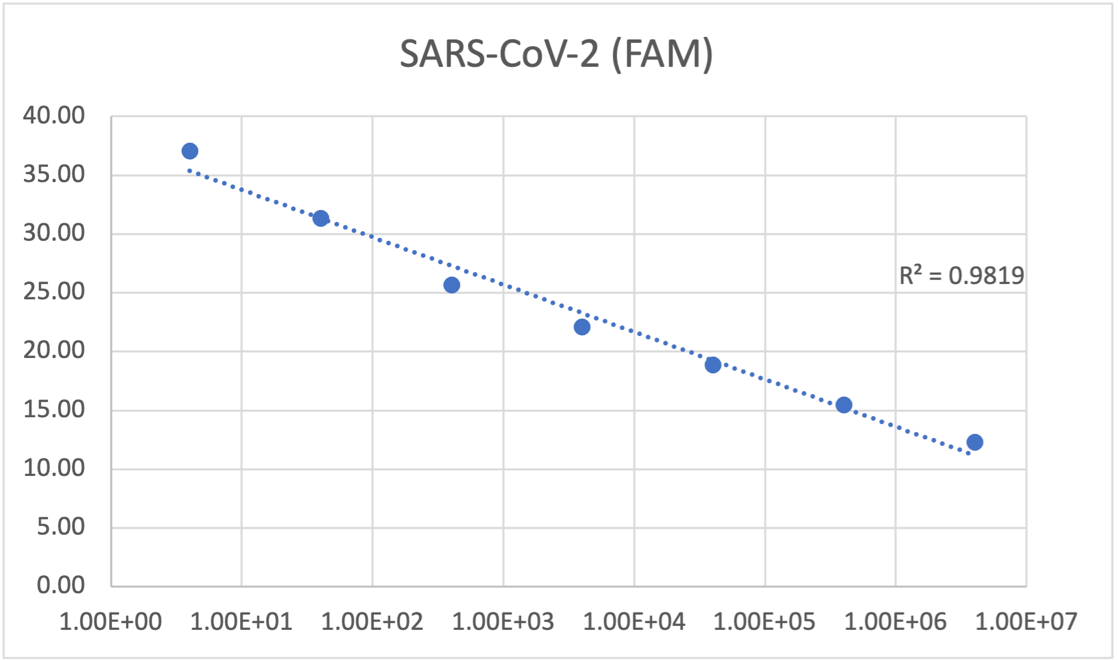


**Figure 1:** Standard curve for limit of detection of SARS-CoV-2. Primers targeted at the Nucleocapsid (N1) gene have a calculated efficiency of 98.02%.


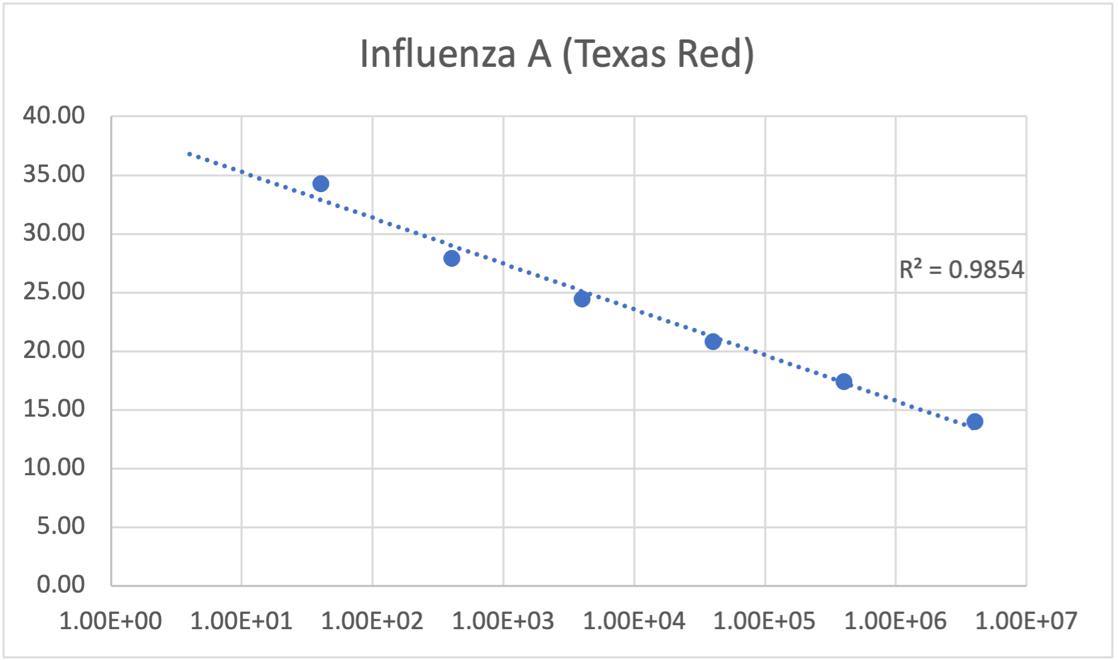


**Figure 2:** Standard curve for limit of detection of Influenza A (H1N1). Primers targeted at the matrix gene have a calculated efficiency of 80.45%.


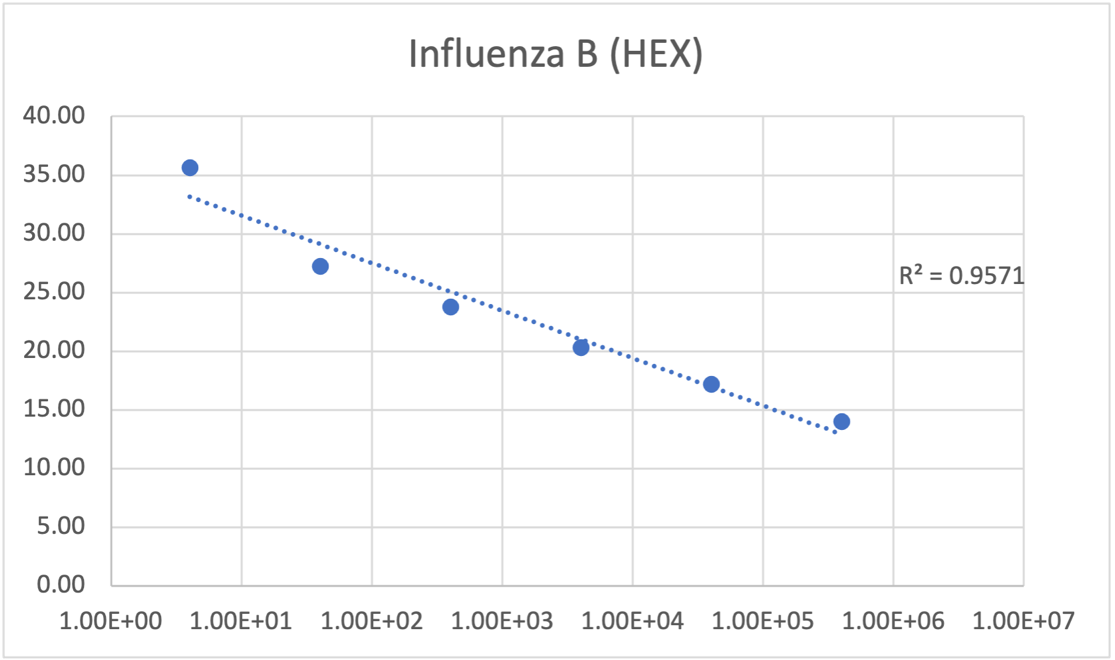


**Figure 3:** Standard curve for limit of detection of Influenza B. Primers targeted at the non-structural protein gene have a calculated efficiency of 90.65%.
